# The Venom of *Ornithoctonus huwena* affect the electrophysiological stability of neonatal rat ventricular myocytes by inhibiting sodium, potassium and calcium current

**DOI:** 10.1080/19336950.2018.1449497

**Published:** 2018-04-05

**Authors:** Sha Yan, Pengfei Huang, Ying Wang, Xiongzhi Zeng, Yiya Zhang

**Affiliations:** a Department of Dermatology, Xiangya Hospital, Central South University, Changsha, China; b Key Laboratory of Organ Injury, Aging and Regenerative Medicine of Hunan Province, Central South University, Changsha, Hunan, China; c The Key Laboratory of Protein Chemistry and Developmental Biology of Ministry of Education, College of Life Sciences, Hunan Normal University, Changsha, P. R. China; d The National and Local Joint Engineering Laboratory of Animal Peptide Drug Development, College of Life Sciences, Hunan Normal University, Changsha, China

**Keywords:** action potential duration, cardiac ion channels, *O. huwena*, spider venom, ventricular myocytes

## Abstract

Spider venoms are known to contain various toxins that are used as an effective means to capture their prey or to defend themselves against predators. An investigation of the properties of *Ornithoctonus huwena (O.huwena)* crude venom found that the venom can block neuromuscular transmission of isolated mouse phrenic nerve-diaphragm and sciatic nerve-sartorius preparations. However, little is known about its electrophysiological effects on cardiac myocytes. In this study, electrophysiological activities of ventricular myocytes were detected by 100 μg/mL venom of *O.huwena*, and whole cell patch-clamp technique was used to study the acute effects of the venom on action potential (AP), sodium current (I_Na_), potassium currents (I_Kr_, I_Ks_, I_to1_ and I_K1_) and L-type calcium current (I_CaL_). The results indicated that the venom prolongs APD_90_ in a frequency-dependent manner in isolated neonatal rat ventricular myocytes. 100 μg/mL venom inhibited 72.3 ± 3.6% I_Na_ current, 58.3 ± 4.2% summit current and 54 ± 6.1% the end current of I_Kr_, and 65 ± 3.3% I_CaL_ current, yet, didn't have obvious effect on I_Ks_, I_to1_ and I_K1_ currents. In conclusion, the *O.huwena* venom represented a multifaceted pharmacological profile. It contains abundant of cardiac channel antagonists and might be valuable tools for investigation of both channels and anti- arrhythmic therapy development.

## Introduction

The mammalian heart is a mechanical pump with the function of assuring pulmonary and systemic blood circulation. Excitability of cardiac myocytes is caused by ionic fluxes through a series of activity of diverse ion channels. It is well recognized that the shape and duration of cardiac action potential are determined by a balance (i.e., sequential activation and inactivation) of inward currents and outward currents [1]. The inward currents include voltage-gated Na^+^ current (I_Na_) and and L-type Ca^2+^ current (I_CaL_). I_Na_ is responsible for the phase 0 depolarization and I_CaL_ is responsible for maintaining plateau (phase 2) of the action potential. The outward currents are carried by four prominent K^+^ channels, containing the transient outward K^+^ current I_to1_, the rapidly and slowly activating delayed rectifier K^+^ currents (I_Kr_ and I_Ks_) and the inward rectifier K^+^ current I_K1_, in cardiac ventricular myocytes. These K^+^ currents contribute to repolarization of different phases of the action potential [1-4]. The extent of early repolarization (notch) affects the time course of the other voltage-gated currents and controls the action potential duration (APD) indirectly. The plateau phase depends on a delicate balance of inward (depolarizing) and outward (repolarizing) currents, and the depolarizing force is mainly a Ca^2+^ influx which slowly declines as L-type calcium channels inactivate, but also non-inactivating Na^+^ current can support the plateau phase [5]. The repolarizing action depends on K^+^ efflux due to activation of several voltage-gated potassium channels.

Spider venoms comprise a mixture of compounds with diverse biological activities, which are used as efficient means to capture their prey or to defend themselves against predators. These toxins are of interest as tools for studying neurophysiology and contribute to pharmacology and insecticides [6,7]. Chinese bird spider *O.huwena* (*Ornithoctonus huwena Wang*) is distributed in the hilly area of Yunnan and Guangxi in the south of China [8,9]. *O.huwen* is one of the most venomous spiders in China and a female *O. huwena* can kill a mouse or a sparrow in less than 2 min [9]. Previous work showed that *O. huwena* venom as a mixture of compounds includes abundant enzymes, lectins, enzyme inhibitors and ion channel inhibitors, indicating different types of biological activities [9,10]. For example, HWTX-I, HWTX-V and HWTX-X are N-type Ca^2+^ channel antagonist [7,11–14]. HWTX-IV specifically inhibits the neuronal tetrodotoxin-sensitive (TTX-S) voltage-gated sodium channel [15,16]. However, few study focus on the inhibition of *O.huwena* venom on cardiac ion channels.

In this study, we tested the effect of *O.huwena* venom on action potential duration (APD) and ion channels in Neonatal rat ventricular myocytes (NRVMs). Our results showed that 100 µg/mL venom inhibited the cardiac Na^+^, K^+^ and Ca^2+^ currents and prolonged APDs effectively, implying that *O.huwena* venom is the potential resource for treating cardiac disease.

## Materials and methods

### Ventricular myocyte isolation

NRVMs cells were dissociated from ventricles of 1–2 days old neonatal Sprague-Dawley rats using a previously reported method with some modifications [17]. Ventricular parts of neonatal rats were excised and ventricular tissues were minced on ice and treated with trypsin and collagenase and the cells were cultured in Dulbecco's modified Eagle's medium (DMEM)/F-12 culture medium containing 10% fetal bovine serum as reported earlier [18]. The cell were cultured for 2–5 d for ion current recordings as previously described.

### Collection of the venom

Adult female *O.huwena* spiders were kept in plastic pails covered with plastic net and given water daily. The venom was collected by using an electro-pulse stimulator described previously [19].

### Electrophysiological recording

Whole-cell patch-clamp recordings were performed by an Axon 700B patch-clamp amplifier (Axon Instruments, Irvine, CA, USA) as described previously [19]. Patch pipettes with DC resistance of 2–3 MΩ were fabricated from borosilicate glass tubing (VWR micropipettes; VWR Co., West Chester, PA, USA).The Giga-Ohm seal was achieved under the voltage clamp modeand the sAPs were collected under the current clamp configuration. The Tyrode's solution, extracellular buffer and pipette solution for AP, I_to1_, I_K1_, Cs^+^-carried I_Kr_, I_Na_ and L-type calcium current (I_CaL_) were used as our previously described [20].

The action potentials (APs): the voltage clamp mode was used and the APs were collected under the current clamp configuration using an Axon 700B patch-clamp amplifier (Axon Instruments, Irvine, CA, USA). Perforated patch was used to prolong recording stability. Pipette solution contained 120 mM KCl, 1 mM MgCl_2_, 10 mM EGTA, 10 mM Hepes, and 3 mM MgATP at pH 7.2 adjusted with KOH. Amphotericin B (Sigma) at 500 μg/mL was included in the pipette solution. The extracellular buffer is the modified Tyrode's solution containing 140 mM NaCl, 5.4 mM KCl, 1.3 mM CaCl_2_, 0.5 mM MgCl_2_, 5 mM Hepes, and 5.5 mM glucose at pH 7.4 adjusted with NaOH. Recordings were performed at 30°C.

I_to1_ currents: The CdCl_2_ (200-μmol/L) were added in external solutions to block Ca^2+^-currents. Na^+^-current contamination was avoided by using a holding potential (HP) of -40 mV or by substitution of equimolar choline for external NaCl. I_to1_ current was elicited by 300-ms depolarizing steps from a holding potential of −40mV to potentials ranging from −50 mV to +100 mV in 10-mV increments.

I_Ks_ currents: The external Na^+^ was replaced by equimolar choline (126 mM) and the solution was supplemented by 4-AP (5 mM), BaCl_2_ (0.5 mM), CdCl_2_ (0.2 mM), dofetilide (1 μM) and glibenclamide (1 μM) to suppress potential interference of I_Na_, I_to1_, I_K1_, I_CaL_, I_Kr_ and ATP-dependent K^+^ channels (K_ATP_), respectively. I_Ks_ current was defined as the chromanol 293B–sensitive (10 μM) current and was elicited by 3-s depolarizing steps from a holding potential of −50mV to potentials ranging from −50 mV to +100 mV in 10-mV increments.

I_K1_ currents: The external Na^+^ was replaced by equimolar choline (126 mM) and the solution was supplemented by 4-AP (5 mM), chromanol 293B–sensitive (10 μM), CdCl_2_ (0.2 mM), dofetilide (1 μM) and glibenclamide (1 μM) to suppress potential interference of I_Na_, I_to1_, I_Ks_, I_Ca_, I_Kr_ and ATP-dependent K^+^ channels (K_ATP_), respectively. From a holding potential of -40 mV, test pulses from -120 mV to 0 mV (400ms) were applied to cardiomyocytes in steps of 10 mV.

Cs^+^-carried I_Kr_ currents: the pipette solution contained (in mM): 135 mM CsCl, 10 mM EGTA, 5 mM ATP-Mg, and 10 mM HEPES. The pH was adjusted to 7.2 with CsOH. The bath solution contained (in mM): 135 mM CsCl, 10 mM HEPES, 10 mM glucose, and 1 mM MgCl_2_. 10 μm nifedipine was used to suppress potential interference of I_CaL_. From a holding potential of -80 mV, depolarizations in 10-mV increments to voltages between -70 and +70 mV for 1.5 s were applied to evoke currents.

I_Na_ currents: A low-sodium extracellular solution containing (in mM): 20 mM NaCl, 1 mM MgCl_2_, 1 mM CaCl_2_, 0.1 mM CdCl_2_, 20 mM HEPES, 117.5 mM CsCl, 11 mM glucose, 11. The pipette solution contained (in mM): 5 mM NaCl, 135 mM CsF, 10 mM EGTA, 5 mM MgATP, 5 mM HEPES. To characterize the voltage dependence of the peak I_Na_, single cells were held at -120mV, and 50 ms voltage steps were applied from -100 to +40mV in 10 mV increments. Interval between voltage steps was 3 sec.

L-type calcium current (I_CaL_): the external solution contained (in mmol/L) 136 mM tetraethylammonium chloride (TEA-Cl), 5.4 mM CsCl, 2 mM CaCl_2_, 0.8 mM MgCl _2_, 10 mM HEPES and 10 mM dextrose (pH 7.4 with CsOH). The pipette solution contained (mmol/L) 20 mM CsCl, 110 mM Cs-aspartate, 1 mM MgCl _2_, 5 mM MgATP, 0.1 mM GTP, 10 mM EGTA and 10 mM HEPES (pH 7.2 with CsOH). The I_CaL_ peak was measured repetitively at a test potential of 0 mV for 150 ms from a holding potential of -40 mV, voltage steps were applied from -50 to +50mV in 5 mV increments.

All chemicals and drugs were purchased from Sigma-Aldrich (St. Louis, MO, U.S.A.). chromanol 293B were dissolved in dimethyl sulfoxide (DMSO) to a stock solution of 10 mM and stored at −20 °C. Glibenclamide were dissolved in 70% ethanol to a stock solution of 10 mM and were stored at 4°C. The drugs were diluted in the bathing solution on the day of the experiment. The final concentration of DMSO was < 1%, and DMSO at this concentration had no effect on membrane current. Vehicle control experiments with ethanol or DMSO in a final concentration of 0.1% did not reveal any effects on currents measured with rat ventricular myocytes.

### Data analysis

Patch-clamp data were processed in Clampfit 10.0 and then analyzed in Excel and Origin 9.0. Data for voltage-dependence of activation were fitted to the Boltzmann equation: Y = 1/{1+exp[2(V _m_-V_1/2_)/*K*]}, where V_m_ is the membrane potential, V_1/2_ is the half-activation or half-deactivation potential, and *K* is the inverse slope factor (in mV) reflecting the steepness of the voltage dependence of gating. For G-V curves, Y represents the relative conductance (G/G_max_). Data are given as means ± SE. All experiments were performed at room temperature (23 ± 0.1 °C), except for AP recordings being performed at 30 °C.

## Results

### The venom prolongs APD in isolated neonatal rat ventricular myocytes

**Figure 1.**
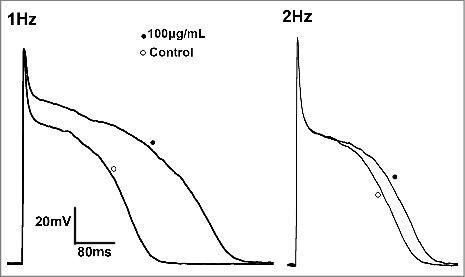
Effect of the venom on action potentials in NRVMs. The action potentials elicited at 1 Hz (left) and (right) in the absence (control) or presence of 100 μg/mL venom.

To test the effect of the venom on action potential duration (APD) in isolated neonatal rat ventricular myocytes, APDs before and after the treatment of 100 μg/mL venom were determined (Figure 1). The data revealed that the venom prolonged APDs of NRVMs in Table 1. Treatment with the venom prolonged both APD_90_ and APD_50_ in ventricular myocytes at the frequency of 1 Hz. APD _90_ increased from 259.2 ± 12.1 ms to 398.3 ± 14.5 ms (*P* < 0.01), while APD_50_ increased from 190.4 ± 11.2 ms to 312.7 ± 10.6 ms (*P* < 0.01). Notably, action potential amplitude (APA) did not change after the venom addition and washout (Table 1). At the frequency of 2Hz, the venom showed less efficiency with the APDs increasing from 241.1 ± 11.2 ms to 272.3 ± 11.2 ms (*P* < 0.01) and APD_50_ increasing from 175.1 ± 8.6 ms to 201.6 ± 10.1 ms. ∆APD _90-50_ is the difference between APD_90_ and APD_50_ recorded at a constant frequency (∆APD_90–50_ = APD _90_ – APD_50_). 100 μg/mL venom greatly prolonged ∆APD_90–50_ to 85.6 ± 10.3 ms compared with the control (∆APD _90–50_ = 68.8 ± 12.2 ms) at the frequency of 1 Hz.

**Table 1.** Effect of the venom (100 μg/mL) on action parameters recorded in rat ventricular myocytes at the frequency of 1 Hz and 2Hz.

	APA (mV)	APD_90_ (ms)	APD_50_ (ms)	∆APD _90–50_ (ms)
2Hz	121 ± 5.7	241.1 ± 11.2	175.1 ± 8.6	66 ± 7.9
2Hz-venom	121 ± 5.7	272.3 ± 11.2^*^	201.6 ± 10.1	70.7 ± 11.1
1Hz	124 ± 6.2	259.2 ± 12.1	190.4 ± 11.2	68.8 ± 12.2
1Hz-venom	124 ± 6.2	398.3 ± 14.5^*^	312.7 ± 10.6^*^	85.6 ± 10.3^*^

APA: action potential amplitude. APD_90_: 90% of action potential duration. APD_50_: 50% of action potential duration.

* P<0.01 in paired t-test, compared with the baseline (n = 10).

### Effects of the venom on I_Na_ in isolated neonatal rat ventricular myocytes

**Figure 2.**
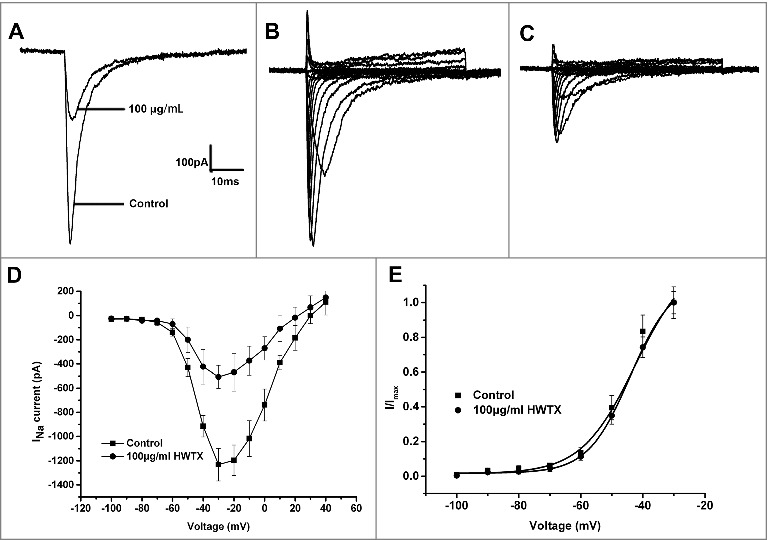
Effect of the venom on I_Na_ currents recorded in neonatal rat ventricular myocytes. Currents were elicited by voltage steps from a holding potential of −120 mV. (a) 100 µg/mL venom inhibited I_Na_ currents. (b) and (c) Representative recording of whole cell currents in the absence or presence of the venom. (d) and (e) Effect of the venom on average steady-state current–voltage (I–V) relationship and G-V relationship.

Nav1.5, as the main voltage-gated sodium channel on ventricular myocytes, generates the fast depolarization of the cardiac action potential and plays a key role in cardiac conduction [19]. I_Na_ was elicited by pulses to -30 mV from a holding potential of -120 mV in in rat ventricular myocytes. As shown in Figure 2, 100 μg/mL venom strongly inhibit cardiac I_Na_ currents by 72.3 ± 3.6%, (n>8), indicating that cardiac I_Na_ channels antagonists indeed existed in the venom. The current-voltage (I-V) curves before and after the venom treatment showed that the inhibition did not associate with evident changes in the I-V relationships of the cardiac I_Na_ currents (Figure 2B, C and D). The venom treatment did not alter the the voltage dependence of cardiac I_Na_ channel activation (the half-maximal activation potential (V_1/2_) = -41 ± 0.4 mV for control and V _1/2_ = −43 ± 0.9 mV for 100 μg/mL venom) in Figure 2E. Our data indicated that it was possible to identify I_Na_ antagonists with therapeutic potentials from the venom of *O.huwena*.

### The venom of *O.huwena* effects on I_to1_, I _K1_, I_Ks_ and I_Kr_ ventricular repolarizing currents

Ventricular myocytes K^+^ channels contribute to the regulation of ventricular repolarization, including transient outward K^+^ current (I_to1_), the rapid (I_Kr_) and slow (I_Ks_) components of the delayed rectifier current and the inward rectifier current (I_K1_). Here, we analyzed the effects of *O.huwena* venom on ventricular myocytes K^+^ currents.

**Figure 3.**
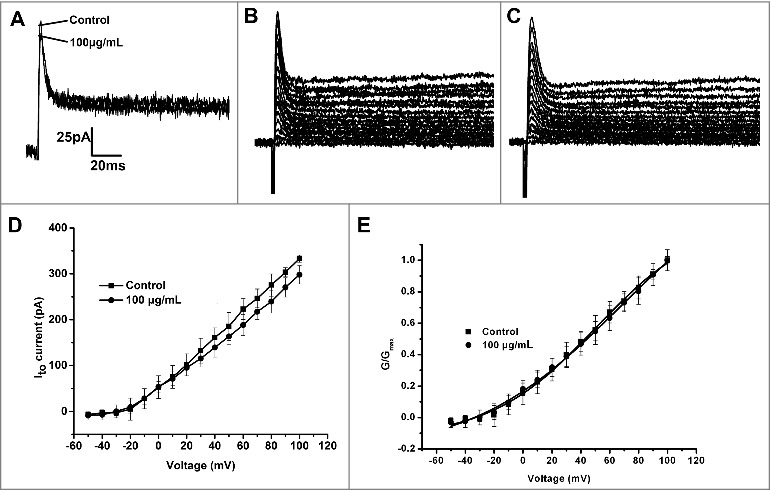
Effect of the venom on I_to1_ currents recorded in neonatal rat ventricular myocytes. Currents were elicited by voltage steps from a holding potential of −40 mV. (a) 100 µg/mL venom inhibited I_to1_ currents. (b) and (c) Representative recording of whole cell currents in the absence and presence of the venom (100 µg/mL). (d) and (e) Effect of the venom on average steady-state current–voltage (I–V) relationship and G-V relationship.

I_to1_ is responsible for the initial rapid repolarization (phase 1) and determines the height of the early plateau, thus influencing the activation of other currents that control repolarization, mainly including I_CaL_ and the delayed rectifier K^+^ currents (I_K_) [2]. As shown in Figure 3A, 100 μg/mL venom did not inhibited the outward peak currents of I_to1_ evidently (6.8 ± 4.1%) and not affect the half-maximal activation potential (V_1/2_), ranged from 26.5 ± 0.3 Mv in control to 27.1 ± 0.4 mV in the presence of the venom. It may indicate that the venom has no significant effect on the activation of I_to1_ current in rat ventricular myocytes (Figure 3B, C, D and E).

**Figure 4.**
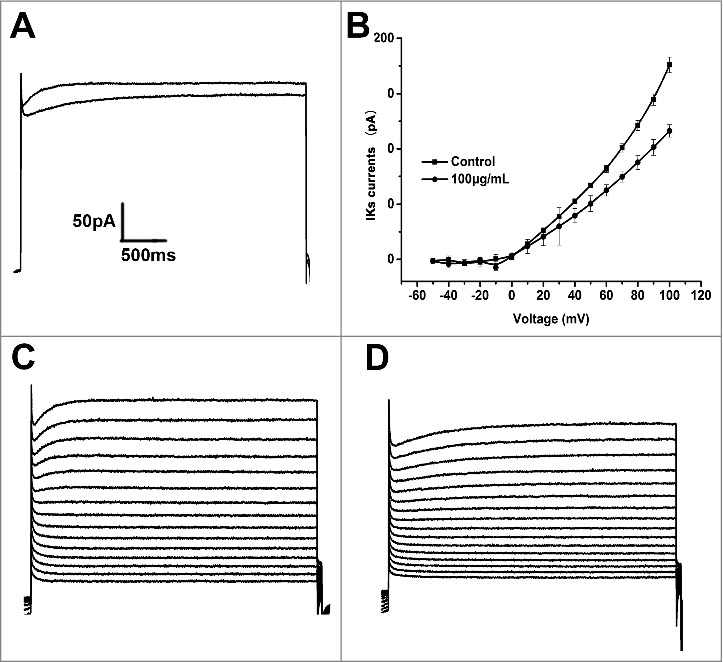
Effect of the venom on I_Ks_ currents recorded in neonatal rat ventricular myocytes. Currents were elicited by voltage steps from a holding potential of −40 mV. (a) 100 µg/mL venom inhibit I_Ks_ currents. B Effects of the venom on average steady-state current–voltage (I–V) relationship. (b) and (c) Representative recording of whole cell currents in the absence and presence of the venom (100 µg/mL).

IK comprises two distinct current components: slowly activating delayed rectifier outward K^+^ currents (I_Ks_) and rapidly activating delayed rectifier outward K^+^ currents (I_Kr_). Here, class III antiarrhythmic agent dofetilide (1 μM), a selective blocker of I_Kr_, were used to inhibit I_Kr_ and then I_Ks_ was recorded independently. Figure 4A showed the I_Ks_ assessment in NRVMs by a 3-s-long voltage-clamp pulse protocol. The slowly developing outward current was exhibited in Figure 4B. As shown in Figure 4C and 4D, even administrated with the venom at the concentration of 100 μg/mL, there was no substantive change in I_Ks_ with an inhibition of 8.3 ± 4.4%, without changing the I-V curves.

**Figure 5.**
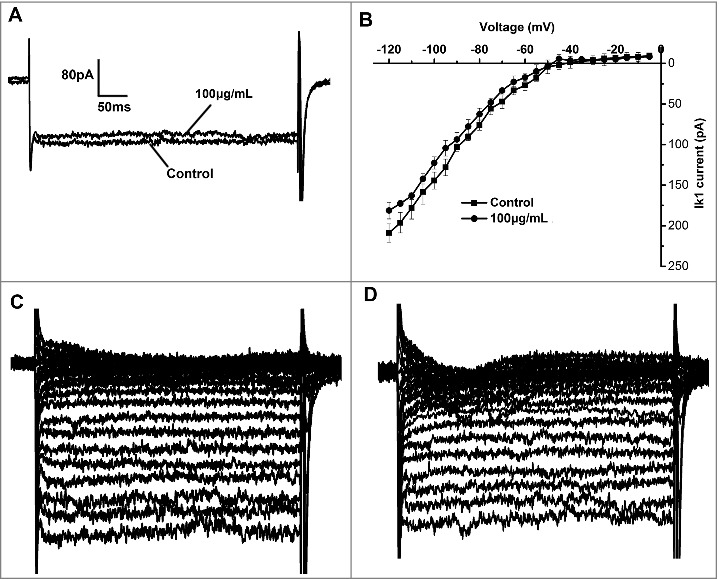
Effect of the venom on I_K1_ currents recorded in neonatal rat ventricular myocytes. Currents were elicited by voltage steps from a holding potential of −40 mV. (a) 100 µg/mL venom inhibit I_K1_ currents. (b) Effects of the venom on average steady-state current–voltage (I–V) relationship. (c) and (d) Representative recording of whole cell currents in the absence and presence of the venom (100 µg/mL).

It has been reported that inward rectifying K^+^ current (I_K1_) diminishes in the adult heart failure, which is the onset of arrhythmias. The strong I_K1_ is critical for stabilizing the membrane potential in ventricular myocytes. Raw traces before and after 100 μg/mL venom treatment were shown in Figure 5A. An acute application of the venom had slight effect on I_K1_ in NRVMs, leading to approximately about 10.4 ± 5.2% (n >5) reduction of I _K1_ currents, no changes of the half-maximal activation potential (V_1/2_) were observed (Figure 5B, C and D).

**Figure 6.**
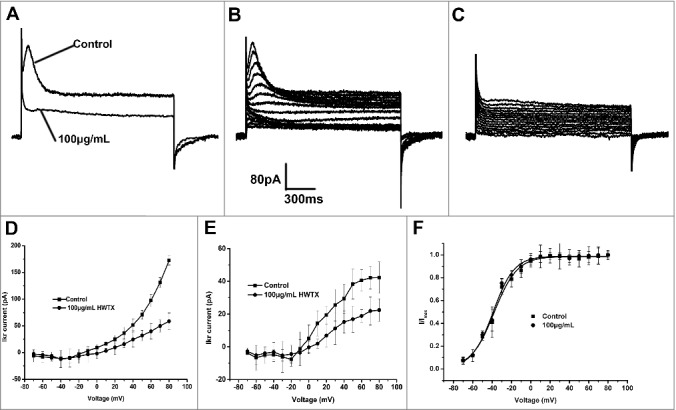
Cs^+^ currents recorded in rat ventricular myocytes with both pipette and bath solutions containing 135 mM Cs^+^. (a) The Cs^+^ currents elicited by depolarization to voltages +50 mV from the holding potential -80 mV in the absence (control) and presence of 100 μg/mL venom. (b) and (c) Representative recording of whole cell currents in the absence and presence of the venom (100 µg/mL). (d) Effects of the venom on average steady-state current–voltage (I–V) relationship of the maximal current during depolarization. (e) Effects of the venom on average steady-state current–voltage (I–V) relationship of the current at the end of depolarizing steps. (f) Effects of the venom on average steady-state current–voltage (I–V) relationship of the tail current. Amplitudes of the tail currents on repolarizations to -70 mV and were normalized to the largest tail current and plotted against depolarizing voltages. Data were fitted to a Boltzmann function.

E-4031-sensitive I_Kr_ is small, and its recording represents a tedious task. Previous data showed that hERG and I_Kr_ channels display unique Cs^+^ permeability [21]. We recorded the pure I_Kr_ in neonatal rat ventricular myocytes using isotonic Cs^+^ solutions (135 mM Csi+/135 mM Cso+) as described previously [21,22]. Figure 6 showed that a family of Cs^+^ currents obtained from a single cardiomyocyte. From a holding potential of -80 mV, depolarizations in a 10-mV increments to voltages between -70 and +80 mV for 1.5 s were applied to evoke currents. Depolarizing steps to voltages above 0 mV induced outward currents, which inactivated in a voltage-dependent manner. The following tail currents at -80 mV displayed an initial rising phase, which is usually described as a “hook,” reflecting the rapid recovery of inactivated channels to the open state before deactivation, and is unique to I_Kr_ [23]. Figure 6A showed that Cs^+^ carried I_Kr_ recorded from cardiomyocyte before and after the application of 100 μg/mL venom and the venom inhibited peak currents, currents at the end of 1-s depolarizing steps and the tail currents by 58.3 ± 4.2%, 54 ± 6.1% and 8.3 ± 3.7%, respectively. The I-V relationships of peak currents and currents at the end of 1-s depolarizing step were shown in Figure 6D and E, and the tail current activation curves were showed in Figure 6F before and after the application of the venom. 100 μg/mL venom did not affect the half-maximal activation potential (V_1/2_) of tail currents (from -38.2 ± 1.7 mV in control to -38.8 ± 1.4mV in the presence of the venom).

In conclusion, our study showed that the venom inhibited I_Kr_ significantly and inhibited other cardiac potassium currents (I_to1_, I_Ks_ and I_K1_) slightly.

### Effects of the venom on I_CaL_ in isolated neonatal rat ventricular myocytes

**Figure 7.**
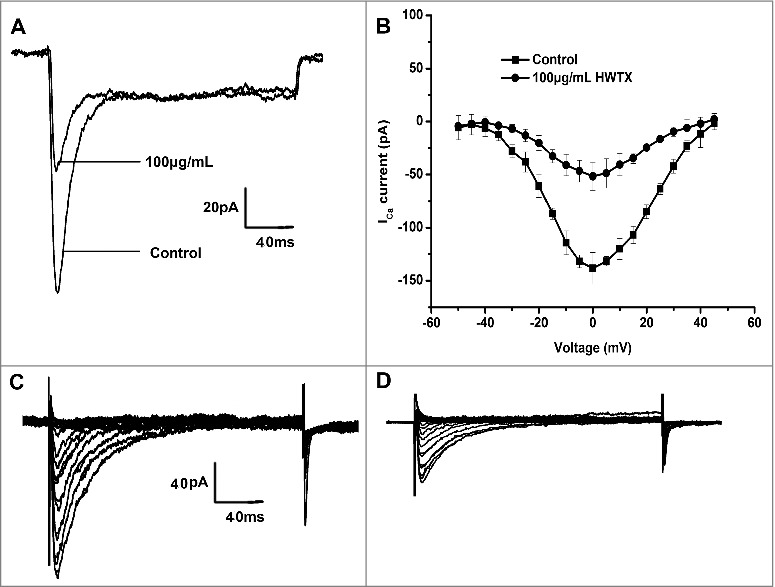
Effect of the venom on I_CaL_ currents recorded in neonatal rat ventricular myocytes. Currents were elicited by voltage steps from a holding potential of −40 mV. (a) 100 µg/mL venom inhibited I_CaL_ currents. (b) Effects of the venom on average steady state current–voltage (I–V) relationship. (c) and (d) Representative recording of whole cell currents in the absence and presence of *O.huwena* venom (100 µg/mL).

Because Ca^2+^ currents (I_CaL_) are increased during hypertrophy and heart failure [23], we examined the effects of the venom of *O.huwena* on I_CaL_ in NRVMs. The I_CaL_ current was evoked at a test potential of 0 mV for 150 ms from a holding potential of -40 mV. Typical L-type I_Ca_ recordings before and after venom treatment are shown in Figure 7A. 100 μg/mL venom decreased I_CaL_ currents by 65 ± 3.3%, without any alteration in the form of the I-V curve (Figure 7 B, C and D).

## Discussion

The mammalian heart is a mechanical pump with the function of assuring pulmonary and systemic blood circulation. Six prominent voltage-gated ion currents expressed in cardiac ventricular muscle are sodium current (INa), rapid activating delayed rectifier outward K+ current (IKr), slowly activating delayed rectifier outward K+ current (IKs), transient outward K+ current (Ito1), inward rectifier K+ current (IK1) and L-type calcium current (ICaL) [24]. These currents contribute in a precisely timed and regulated manner to the development, maintenance and termination of the action potential [25]. In this study, our work increased the knowledge about the electrophysiological effect of O.huwena venom on the action potential (AP), cardiac Na+, K+ and Ca2+ channels.

The O.huwena venom prolonged both APD90 and APD50 in ventricular myocytes at both 1 Hz and 2 Hz, but the effect of the venom at 1 Hz frequency is more effectively than 2 Hz. The similar result also observed in Verkerk's study which showed that DIDS (4,4′diisothiocyanatostilbene-2,2′-disulphonic acid) increased APD only at 3.33 Hz, but not at the lower stimulus frequencies [26]. Moreover, dofetilide and 293B also showed different effects on action potential at different Hz duration [27]. Given the pronounced effects of O.huwena venom on APD prolongation, which ion channels are responsible for this action was an essential question to address. In this study, 100 μg/mL venom strongly inhibit cardiac INa、IKr and ICaL currents by 72.3%, 58% and 65% respectively. These results indicated that the venom may contain peptides which have high affinity on cardiac Na、Kr and CaL channels. Previous studies showed that dofetilide, a class III antiarrhythmic drug, was recommended for the treatment of persistent atrial fibrillation the rapid component of the outward delayed rectifier potassium current IKr specifically [28]. Azimilide blocks the slow (I(Ks)) and fast (I(Kr))、sodium (I(Na)) and calcium currents (I(CaL)), it has antiarrhythmic effects to prevent reentry causing sustained ventricular tachycardia (SVT) and ventricular fibrillation (VF) initiation [29]. Therefore, the O.huwena venom could contain toxin peptides as the potential antiarrhythmic drug.

Cardiac INa contributes to initial rapid repolarization in AP, the inward depolarizing currents (ICaL) plays an important role on the balance of plateau phase in AP. We found that O.huwena venom inhibited the two major inward currents generating the ventricular AP, without affecting the upstroke phase. Although Phase 0 is defined by the activation of voltage-dependent Na+ channels giving rise to inward movement of Na+, the inhibitors of Na+ currents did not affect the AP as various studies described [30]. Efonidipine, a Ca2+ channel blockers, reduced arrhythmias in a mouse model of dilated cardiomyopathy by repolarizing the resting membrane potential [31]. In this study, O.huwena venom effectively inhibited cardiac Na+, K+ and Ca2+ channels, and lead to AP prolongation. One possibility is that the inhibition of the K+ currents, especially the 58.3% inhibition of IKr currents could have a greater net effect on the AP duration than the inhibition of ICaL. Another possibility is that the crude venom affects other targets in ventricular cells that have not been tested by patch clamping. As described in previous work, the venom of spider showed current inhibition on cardiac sodium channels, potassium channels and calcium channels [20]. The Chinese tarantula O.huwena is similar to the spider O. hainana in morphology, and the toxin peptides in their venom gland showed high sequence homology [32,33]. However, divergences of electrophysiological effect on cardiac ion channels were observed between two spider venom. In this study, O.huwena venom exhibited more inhibitory activity against cardiac sodium currents and cardiac potassium currents (IKr) compared with O.hainana venom. Moreover, the inhibitory activity of O.huwena venom on cardiac calcium currents was only 65%, which is much less than O.hainana venom.

In conclusion, our work increased the knowledge about the electrophysiological effect of O.huwena venom on the action potential (AP), cardiac Na+, K+ and Ca2+ channels, and indicated that spider venom, containing abundant of cardiac channel antagonists, might be valuable tools for investigation of both channels and anti- arrhythmic therapy development.
